# Dispatcher-Assisted Cardiopulmonary Resuscitation: Disparity between Urban and Rural Areas

**DOI:** 10.1155/2020/9060472

**Published:** 2020-06-01

**Authors:** Yen-Chin Chen, Shao-Hua Yu, Wei-Jen Chen, Li-Chi Huang, Chih-Yu Chen, Hong-Mo Shih

**Affiliations:** ^1^Department of Emergency, China Medical University Hsinchu Hospital, Hsinchu, Taiwan; ^2^Department of Public Health, China Medical University, Taichung, Taiwan; ^3^Department of Emergency Medicine, China Medical University Hospital, Taichung, Taiwan; ^4^Graduate Institute of Biomedical Sciences, China Medical University, Taichung, Taiwan; ^5^Emergency Medical Technician-Paramedic, Fire Bureau of Taichung City Government, Taichung, Taiwan; ^6^School of Nursing, China Medical University, Taiwan Adjunct Supervisor, China Medical University Hospital, Taichung, Taiwan

## Abstract

**Methods:**

Patients with out-of-hospital cardiac arrest (OHCA) were prospectively registered in Taichung. The 29 districts of Taichung city were divided into urban and rural areas based on whether the population density is more than 1,000 people per square kilometer. Prehospital data were collected according to the Utstein-style template, and telephone auditory records were collected by a dispatch center.

**Results:**

2,716 patients were enrolled during the study period. 88.4% OHCA occurred in urban areas and 11.6% in rural areas. 74.9% after dispatcher assistance, laypersons performed CPR in urban areas and 67.7% in rural areas (*p*=0.023). The proportion of laypersons continued CPR until an emergency medical technician's (EMT) arrival was higher in the urban areas (59.57% vs 52.27%, *p*=0.039). Laypersons continued CPR until an EMT' arrival would increase the chance of return of spontaneous circulation in urban and rural areas, with adjusted odds ratio (aOR) of 1.02, 95% confidence interval (CI) of 0.82–1.27, and aOR of 1.49, 95% CI of 0.80–2.80, respectively. Continued laypersons CPR until the EMT' arrival also improved survival with favorable neurological function, with aOR of 1.16, 95% CI of 0.61–2.20 in urban areas and aOR of 2.90 95% CI of 0.18–46.81 in rural areas.

**Conclusion:**

Bystanders in urban areas exhibited higher ratio of acceptance of DACPR. However, after DACPR intervention, prognosis improvement was considerably higher in rural areas than in urban areas.

## 1. Introduction

Approximately 400,000 cases of out-of-hospital cardiac arrest (OHCA) occur in the United States each year, which accounts for 13.5% of the total mortality rate. The incidence of OHCA has been increasing each year [1]. Early recognition of OHCA, early bystander cardiopulmonary resuscitation (CPR), and automated external defibrillation (AED) have substantial effects on survival rates and neurological outcomes in patients with OHCA [1–3]. However, the time gap between the recognition of cardiac arrest to the arrival of emergency medical technicians (EMTs) is long, and the interventions from bystanders before an EMT's arrival could critically affect the prognosis in these patients [4]. A study showed that bystanders do not perform CPR before an EMT's arrival because of panic, concerns about not performing CPR accurately, fear of harming the individual, fear of legal consequences, and concern about mouth-to-mouth contact [5, 6]. The survival rates of patients with OHCA have gradually increased recently through the promotion of the resuscitation training program, simplified CPR process, improved CPR quality by laypersons, and willingness [4].

Dispatcher-assisted CPR (DACPR) encourages laypersons to perform CPR [1, 7, 8]. In Chicago, after establishing integrated resuscitation systems of care, the bystander CPR rate increased from 11.6% to 19.4%, the return of spontaneous circulation (ROSC) rate increased from 28.6% to 36.9%, and the overall survival rate improved from 7.3% to 9.9% [9]. Patients receiving DACPR presented superior prognosis. DACPR has been deemed as an effective method for encouraging laypersons to perform CPR early and improve patient survival [10, 11].

Survival outcomes and neurological outcomes for patients with OHCA varied from 3.4% to 22% and 0.8% to 21% between rural and urban areas, respectively. However, disparities exist in the effects of CPR between rural and urban areas due to their different characteristics, distances, population densities, and resources [12, 13]. Most studies regarding DACPR have focused on the prognosis of patients with OHCA. However, limited studies have discussed the disparities in DACPR effects between rural and urban areas. The aims of this study were to compare the provision and effectiveness of DACPR in rural and urban areas according to different population densities and to make robust suggestions for improving CPR effects.

## 2. Methods

### 2.1. Study Design and Settings

To assess the effectiveness of DACPR in urban and rural areas, we retrospectively analyzed the prospective registered data of patients with OHCA in Taichung City. Taichung City consists of 29 districts, including urban and rural areas. In 2018, Taichung had more than 2.8 million residents, 49 local fire departments, and one dispatch center. More than 2,500 patients with OHCA are referred to emergency medical services (EMS) each year in Taichung. In Taichung, EMTs provided mainly basic life support for patients with OHCA, including airway management with bag-valve-mask and laryngeal mask airway, chest compression, and shock as necessary with AED [14]. EMT needs to be trained 24 hours a year, and the BLS license needs to be renewed every two years. Based on whether the population density is greater than 1,000 people per square kilometer, we divided 29 districts into urban and rural groups.

All 119 call emergency situations, such as emergency medical incidents or fire accidents, are connected to the Taichung dispatch center. The dispatch center is responsible for dispatching personnel and ambulances to the scene of the accident and providing necessary assistance, such as DACPR. Since 2015, DACPR has been promoted in the region. All dispatchers have received at least 8-h training on providing DACPR. After 2 years of running-in, DACPR was implemented gradually across Taichung.

### 2.2. Selection of Participants

All patients who experienced OHCA in Taichung City between 1 July, 2017 and 30 November, 2018 were initially included in the study. Patients who appeared apparently dead, refused hospital referral, aged younger than 18 years, and experienced cardiac arrest following trauma were excluded. Patients with cardiac arrest in medical facilities were also excluded. Those who had cardiac arrest after an EMT' arrival, those who refused transfer to hospital, those who received CPR from a bystander without DACPR guidance, and those who lacked audio file records were also excluded.

### 2.3. Data Collection

Prehospital data were collected according to the Utstein-style template, which included age, sex, witnessed status, bystander CPR, the location where OHCA occurred, and use of AED. Each telephone recording of OHCA cases was collected and analyzed for report time, whether recognition of OHCA, recognition time, time of first chest compression after instruction of CPR, EMS response time (defined as time interval between calling to EMT' arrival at the scene), and whether continued CPR until EMT arrival.

### 2.4. Outcome Measures

The primary outcome of this study was the disparity in bystanders DACPR rate between rural and urban areas after dispatcher's instruction. The secondary outcome was whether the cardiac arrest status was identified, the time taken for the 119 call, identification of the cardiac arrest status, confirmation by dispatchers of bystanders performing chest compression, first chest compression time, and total CPR duration.

Patient outcomes were analyzed including prehospital ROSC, sustained ROSC, and neurological outcomes at discharge. Sustained ROSC was defined as ROSC for more than 2 h. Neurological outcomes were assessed using Cerebral Performance Category (CPC) systems [15]. Favorable neurological outcomes were defined as CPC 1-2 scores with superior cerebral performance or moderate cerebral disability with independent activities of daily living.

### 2.5. Statistical Methods

Patients with OHCA were divided into urban and rural groups according to population densities in each area of Taichung City. For demographic characteristics of the two groups, continuous variables were presented as means ± standard deviation and compared using the independent sample *t*-test. Nominal variables were presented as percentages of the frequency of occurrence and compared using the chi-square test.

We used the chi-square test to compare the recognition rate of patients with OHCA and the DACPR rate after instruction between the urban and rural groups. The time from 119 call to recognition of OHCA, the call to chest compression time, and CPR instruction time were analyzed using the Mann–Whitney *U* test and presented as a median (interquartile range, IQR).

The effect of continued DACPR until EMT arrival on the outcome of patients with OHCA was analyzed through a multivariate logistic regression after adjustment for age, witness, location, AED shock, and EMS response time.

All statistical assessments included the two-tailed test. Statistical significance was defined as *p* < 0.05. All statistical analyses were performed using SAS (version 9.4; SAS Institute, Inc., Cary, NC). This study was approved by the Institutional Review Board of China Medical University.

## 3. Results

**Figure 1:**
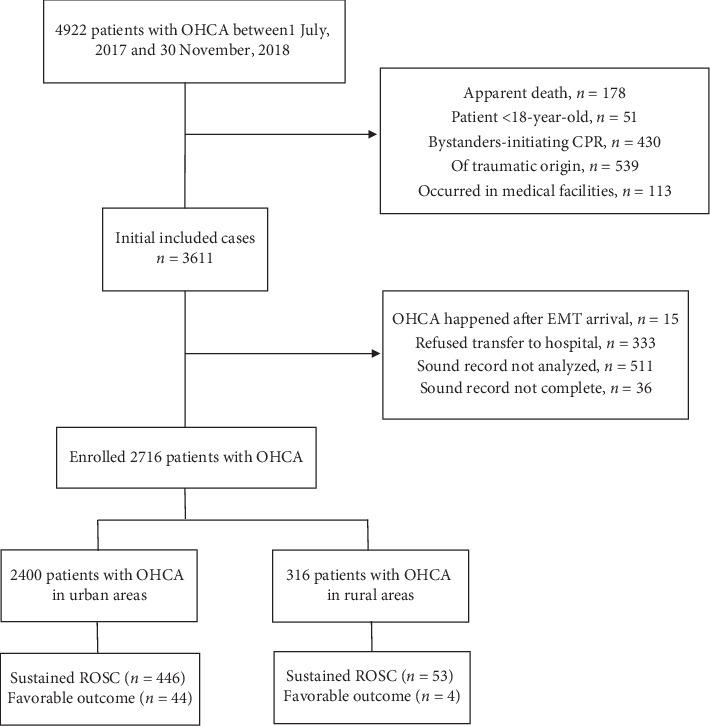
Flow chart of patient enrollment.

**Figure 2:**
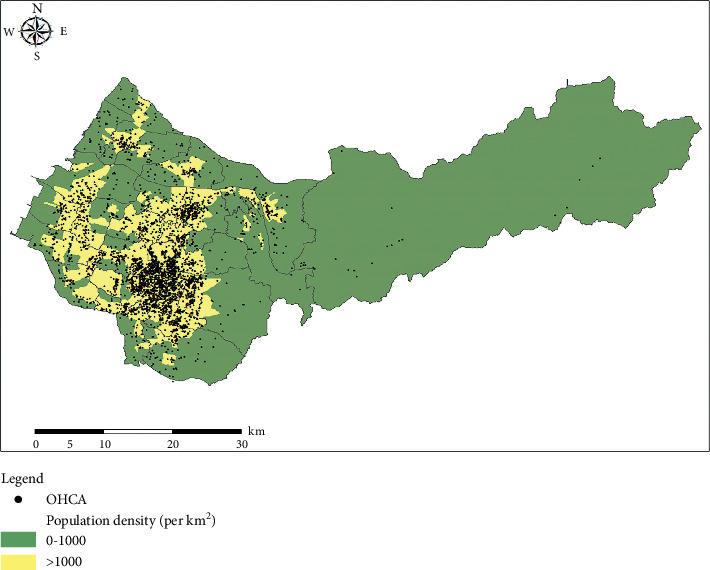
Map of Taichung. Each point represented the location of OHCA events.

A total of 4,922 patients with OHCA called the EMS during the study period. Among them, 2,716 patients were enrolled in this study after excluding those who met the aforementioned exclusion criteria (Figure 1). Approximately 88.4% OHCA occurred in urban areas and 11.6% in rural areas. The location of OHCA occurred and the corresponding population density is shown in Figure 2.

Patients with OHCA in the rural areas were older than those in the urban areas (71.5 ± 16.2 years in urban areas and 73.03 ± 14.9 years in rural areas, respectively; *p*=0.011). The male to female ratio was 6 : 4, and no significant difference was observed between the two groups. Most cases of OHCA occurred at home (79.7%). The proportion of witnessed OHCA was significantly higher in the rural areas than in the urban areas (41.14% vs 33.33%; *p*=0.006). Bystanders performed CPR in 46.7% cases, and 11% cases received AED shock. The average EMS response time was 6.86 min. Compared with urban areas, the EMS response time was longer in rural areas (6.66 ± 2.57 min in urban areas and 8.43 ± 4.68 min in rural areas, respectively; *p* < 0.001).

ROSC rates in urban areas (18.6%) were higher than those in rural areas (16.8%). The survival with favorable neurological function rate was 1.77% in total, with 1.83% in the urban areas and 1.27% in the rural areas, respectively. No difference was observed in ROSC, initial shockable arrests, and survival with favorable neurological function between the urban and rural groups (Table 1).

The rate of OHCA recognition in this study was 68.3%; the median recognition time was 79.9 seconds (s); and no significant difference was observed between the two groups. When patients with OHCA were recognized and DACPR was instructed, 74.9% laypersons performed CPR in urban areas and 67.7% in rural areas (*p*=0.023). The rates of laypersons continuing CPR until an EMT' arrival in recognized OHCA patients were higher in urban areas than in rural areas (59.57% vs 52.27%, *p*=0.039) (Table 2).

Patients with OHCA in urban areas had a relatively higher chance of achieving sustained ROSC. Additionally, a relatively higher proportion of patients with OHCA survived with a favorable neurological function in urban areas; however, the outcomes were not statistically significant (Table 1).

For assessing the effect of continued DACPR until the EMT' arrival on the outcome of patients with OHCA, we performed a multivariate logistic regression. After adjusting for age, witness of collapse, location, defibrillation with AED, and EMT arrival time, we found that continued CPR until the EMT' arrival would increase the adjusted odds ratio for ROSC to 1.02 (0.82–1.27) in urban areas and to 1.49 (0.80–2.80) in rural areas. Continued CPR until the EMT' arrival also improved survival with a favorable neurological function with adjusted odds ratios of 1.16 (0.61–2.20) in urban areas and 2.90 (0.18–46.81) in rural areas (Table 3). The supplementary figure shows the adjusted odds ratio for all factors in the final model and their corresponding 95% confidence intervals (Supplementary file).

## 4. Discussion

In this study, we explored the urban–rural gap of DACPR from a single dispatch center. The DACPR execution rate was higher in urban areas than in rural areas. The study also showed that patients with OHCA may have superior prognosis if they receive continued DACPR until the EMT' arrival in rural areas.

After DACPR guidance, the rates of bystander CPR in urban areas were higher than those in rural areas (74.86% vs 67.73%, *p*=0.023). Lee et al. compared emergency medical dispatch services across Pan-Asian countries, which showed differences in the dispatch service recognized and compression started rate in different countries. The rates varied from 84.1% to 11.8% in different countries and different cities, which also divided into urban, suburban, and rural area according to the population density [16]. The rates of recognition of OHCA by dispatchers were nearly 70% in both the groups, and the median recognition time was 79.7 s. The result was similar to those of a study conducted by Viereck et al. in 2017 [3]. Under the same dispatch center intervention and instruction, urban citizens were more willing and less panicked to perform CPR than rural citizens. This may be attributed to urban citizens being younger in Taichung [17], which is similar to that observed in previous studies [3, 5,12, 13, 18]. Additionally, the government implemented resuscitation curriculum in schools. Our study also showed that patients with OHCA in rural areas had higher ROSC rates and a superior favorable neurological function when they received continued DACPR; the adjusted odds ratio was also higher than that in patients in urban areas. We should strengthen the promotion of bystander CPR, particularly in rural areas.

The EMS response time in rural areas was longer than that in urban areas (8.4–6.7 min, *p* < 0.001) in this study, which was similar to the results of a previous study [13]. The average jurisdiction area of the fire department in the urban areas is 10.9 km^2^ and in the rural areas is 133.5 km^2^ in Taichung. The larger the area, the longer the EMT' arrival time. A previous study also demonstrated that the mortality rates were different within the urban-rural gap, and the main reason was EMT' arrival time [19]. When the EMT' arrival time increased, patient survival rates decreased even after DACPR intervention [20]. In our study, no significant difference was observed in the rates of ROSC and survival discharge between the urban and rural areas. This may be attributed to the high accessibility to health care in Taichung City. Although there are urban and rural areas in Taichung City, the total area of this city is only 2,214 km^2^, and there are 19 hospitals in Taichung City that can handle emergency cases.

The patients with OHCA in rural areas are elder than in urban areas. According to the data from Civil Affairs Bureau of Taichung City, the elderly population ratio (>65-year-old) in urban areas is 12.3% and 16.2% in rural areas. Young people immigrated to urban areas for jobs caused the average ages in rural areas be older [17]. Most incidents of OHCA occurred at home with an average of 80% of total cases in our study, which is similar to the result of previous studies [2, 3, 9, 21]. Patients with OHCA in rural areas had a significantly higher ratio being witness than those in urban areas, and this may be because the daily activity range of elders in the countryside was higher, and they had more frequent communications with neighbors, which led to increased chances of being witnessed when events occurred. Studies have shown that the first bystander of patients with OHCA was their family; however, when family members witnessed an OHCA, the ratio of family members providing CPR was lower than that of nonfamily members [18, 22]. DACPR-instructed family members providing CPR could improve neurological outcomes in patients with OHCA [23]. Because majority of OHCA events occur at home, when propagating the resuscitation policy, the government should emphasize the significance of CPR to family members in order to motivate laypersons to provide CPR.

## 5. Limitations

There were several limitations in this study. First, this was a single observational study; therefore, the causal relationship between the variables could not be explained. Second, the study was limited to Taichung City, which is geographically different from other cities; therefore, the results cannot be generalized to the entire country. The case numbers were limited due to collection time and areas. This study distinguished urban and rural areas by population density under a single dispatch center; however, the study also limited the number of population and regions because of a single administrative region. In the future, it is recommended to use national data analysis to further compare the difference in DACPR. The screening process was subject to selection bias because certain recording files were incomplete or could not be analyzed.

## 6. Conclusions

This study found that in the same dispatch center, bystanders in urban areas exhibited a higher rate of acceptance and performance of CPR. Although no statistically significant differences were observed, patients with OHCA in urban areas had superior prognosis. However, after DACPR intervention, prognosis improvement was higher in rural areas than in urban areas. Public health and education are warranted to encourage people to perform CPR and achieve higher bystander CPR rates, particularly in rural areas.

## Figures and Tables

**Table 1:** Demographic data of patients with OHCA in urban and rural areas.

Variables	Overall	Urban (*n* = 2400)	Rural (*n* = 316)	*p* value
*∗*Age, mean	70.75 ± 16.04	70.46 ± 16.16	73.03 ± 14.85	0.011
Gender				0.594
Male	1704 (62.74)	1502 (62.58)	202 (64.13)	
Female	1011 (37.22)	898 (37.42)	113 (35.87)	
Location				0.307
Home	2165	1921 (80.04)	244 (77.22)	
Public outdoor	68	62 (2.58)	6 (1.90)	
Public indoor	92	82 (3.42)	10 (3.16)	
Others	391	335 (13.96)	56 (17.72)	
*∗*Witness	930	800 (33.33)	130 (41.14)	0.006
AED shock (yes)	298	259 (10.79)	39 (12.34)	0.407
*∗*EMS response time (min), mean	6.86 ± 2.94	6.66 ± 2.57	8.43 ± 4.68	<0.001
Prehospital ROSC	96	84 (3.50)	12 (3.80)	0.787
Sustained ROSC	499	446 (18.58)	53 (16.77)	0.434
Survival with favorable neurological function	48	44 (1.83)	4 (1.27)	0.471

*∗p* < 0.05. ROSC: return of spontaneous circulation. AED: automated external defibrillator.

**Table 2:** Recognition of OHCA and instruction of DACPR in urban and rural areas.

Variables	Urban (*n* = 2400)	Rural (*n* = 316)	*p* value
Recognition of OHCA	1635 (68.13)	220 (69.62)	0.591
Recognized time (sec), median (IQR)	66.0 (33.0–107.0)	68.0 (27.0–109.0)	0.747
*∗*DACPR/recognized	1224/1635 (74.86)	149/220 (67.73)	0.023
*∗*Continued CPR till the EMT arrive	974/1635 (59.57)	115/220 (52.27)	0.039
Call to chest compression time (sec), median (IQR)	168.5 (133.0–220.0)	171.0 (138.0–212.0)	0.689
CPR instruction time (sec), median (IQR)	88.0 (59.0–132.0)	87.0 (60.0–124.0)	0.546

*∗p* < 0.05. OHCA: out-of-hospital cardiac arrest. CPR: cardiopulmonary resuscitation. IQR: interquartile range. EMT: emergency medical technician.

**Table 3:** Multivariate analysis for the effect of continued DACPR in urban and rural areas.

Variables	Urban			Rural		
	n/N (%)	Crude OR (95% CI)	Adjusted OR (95% CI)	n/N (%)	Crude OR (95% CI)	Adjusted OR (95% CI)
*ROSC*						
No continued CPR till the EMT arrive	214/1264 (16.93)	Reference	Reference	26/183 (14.21)	Reference	Reference
Continued CPR till the EMT arrive	232/1136 (20.42)	1.26 (1.03–1.55)	1.02 (0.82–1.27)	27/133 (20.30)	1.54 (0.85–2.78)	1.49 (0.80–2.80)

*Favorable Neurological Function*						
No continued CPR till EMT arrive	20/1264 (1.58)	Reference	Reference	2/183 (1.09)	Reference	Reference
Continued CPR till EMT arrive	24/1136 (2.11)	1.34 (0.74–2.44)	1.16 (0.61–2.20)	2/133 (1.50)	1.38 (0.19–9.94)	2.90 (0.18–46.81)

Adjusted with age, witness of collapse, location, defibrillated with automated external defibrillator, and emergency medical service response time.

## Data Availability

The data used to support the findings of this study are available from the Fire Bureau of Taichung City Government. Due to legal restrictions imposed by the government of Taiwan in relation to the “Personal Information Protection Act”, data cannot be made publicly available. Requests for data can be sent as a formal proposal to the Fire Bureau of Taichung City Government (https://english.taichung.gov.tw/fire/).

## List of abbreviations

OHCA: Out-of-hospital cardiac arrest
CPR: Cardiopulmonary resuscitation
AED: Automated external defibrillation
EMT: Emergency medical technician
DACPR: Dispatcher-assisted cardiopulmonary resuscitation
ROSC: Return of spontaneous circulation
EMS: Emergency medical service
CPC: Cerebral performance category
IQR: Interquartile range.

## Supplementary Materials

Supplementary Materials Odds ratios and the corresponding 95% confidence interval of parameters that are associated with the return of spontaneous circulation (ROSC). Young age, witness of collapse, collapse at public places, and defibrillated with automated external defibrillator were associated with a better chance of ROSC in the urban area. However, the only witness of collapse was associated with a better chance of ROSC in the rural area.
