# Supplementary figures and images for: Dispatcher-Assisted Cardiopulmonary Resuscitation: Disparity between Urban and Rural Areas
Source: Emerg Med Int. 2020 Jun 1;2020:9060472. doi: 10.1155/2020/9060472 (PMC7285275; doi:10.1155/2020/9060472)

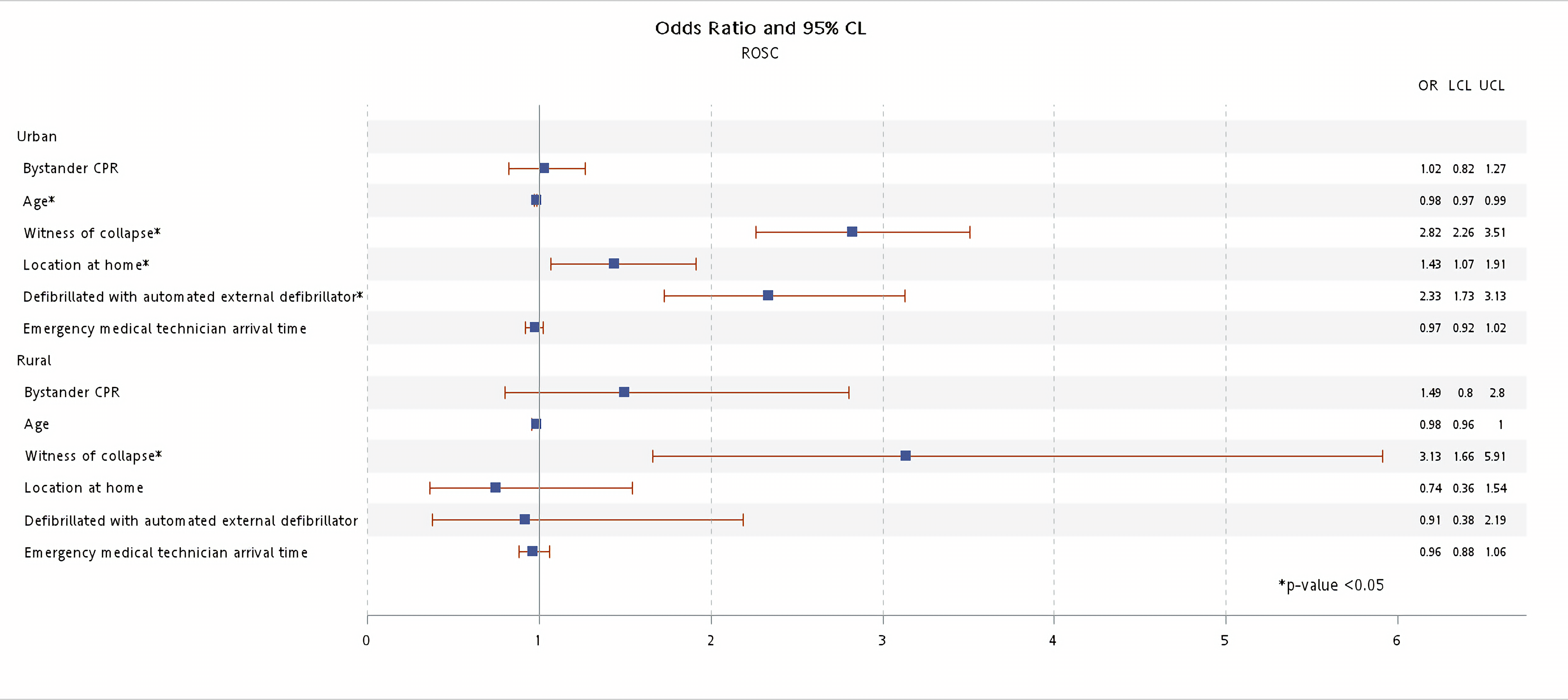

Supplement: Supplementary Materials — Odds ratios and the corresponding 95% confidence interval of parameters that are associated with the return of spontaneous circulation (ROSC). Young age, witness of collapse, collapse at public places, and defibrillated with automated external defibrillator were associated with a better chance of ROSC in the urban area. However, the only witness of collapse was associated with a better chance of ROSC in the rural area. [file 9060472.f1.tif]
